# The complete chloroplast genome sequences of Korean native *Veronica* subgenus *Pseudolysimachion* species (Part I): *V. longifolia* and *V. pusanensis*

**DOI:** 10.1080/23802359.2026.2619288

**Published:** 2026-04-25

**Authors:** Sang Heon Kim, Ji Hun Yi, Jin-Woo Kim, Wonwoo Cho, Ji Young Jung

**Affiliations:** Forest Biological Resources Utilization Center, Korea National Arboretum, Yangpyeong, Republic of Korea

**Keywords:** Chloroplast genome, *Veronica longifolia*, *Veronica pusanensis*, Veroniceae, Plantaginaceae

## Abstract

The genus *Pseudolysimachion* (Plantaginaceae) is currently considered a subgenus of *Veronica*, though its independent status is still debated. In Korea, *Veronica* subgenus *Pseudolysimachion* is diverse, including widespread *Veronica longifolia* (formerly *Pseudolysimachion longifolium*) and endemic *Veronica pusanensis* (formerly *Pseudolysimachion pusanensis*), both valuable for horticulture. Despite chloroplast genome utility in plant phylogenetics and breeding, complete chloroplast genome data for these key species were absent. To address this gap, we sequenced, assembled, and characterized the complete chloroplast genomes of *V. longifolia* and *V. pusanensis*. The complete chloroplast genomes of *V. longifolia* (152,237 bp) and *V. pusanensis* (152,250 bp) displayed typical quadripartite structures and encoded 133 genes (88 protein-coding, 37 tRNA, 8 rRNA). Phylogenetic analysis strongly supported the monophyly of the *Veronica* subgenus *Pseudolysimachion* clade, which is nested deeply within the genus *Veronica*. Our findings provide essential genomic resources that clarify phylogenetic relationships within the subgenus and contribute significantly to the broader taxonomic resolution of the tribe Veroniceae. These results firmly establish the taxonomic positions of the Korean endemic species, *V. longifolia* and *V. pusanensis*, and offer crucial genomic insights into speciation patterns and evolutionary trends within the genus, thereby supporting future plant breeding and germplasm utilization strategies.

## Introduction

The genus *Pseudolysimachion* Opiz (Plantaginaceae) has been separated from *Veronica* L. based on morphological and molecular phylogenetic evidence, yet its taxonomic classification remains contentious due to ongoing complexities in generic delimitation within the tribe Veroniceae (Albach 2008, Albach 2023, Albach et al. 2004). Crucially, recent phylogenetic studies, including those cited, clearly demonstrate that *Pseudolysimahcion* is nested within *Veronica*, leading to its widespread recognition as *Veronica* subgenus *Pseudolysimachion* (Albach 2008, Albach et al. 2004). While morphological traits have historically complicated the delimitation between *Veronica* and its subgenera, complete chloroplast genomes provide high-resolution phylogenetic markers that can clarify these relationships, particularly within the tribe Veroniceae. In Korea, the *Veronica* subgenus *Pseudolysimachion* is represented by 10 native species and 7 infraspecific taxa, accounting for a total of 17 recognized native taxa, which exhibit substantial morphological diversity. Among these species, Long-leaf spike speedwell (*Veronica longifolia* L. 1753) and Busan spike speedwell (*Veronica pusanensis* Y.N.Lee 2004) are two notable native taxa in Korea. While *V. longifolia* exhibits a broad distribution from Europe to the Korean Peninsula (Trávníček 2000), *V. pusanensis* is known to be endemic to the Korean Peninsula (Lee 2005). Given their ornamental potential, both species have been subjects of diverse recent studies.

Chloroplast genomes (plastomes) are highly valuable for resolving phylogenetic relationships and taxonomic ambiguities in plants (Gitzendanner et al. 2018). Comprehensive plastome data are particularly crucial for complex groups like *Veronica* and its segregates, where generic boundaries remain contentious (Albach 2023). Although chloroplast genomes of other *Veronica* subgenus *Pseudolysimachion* species, such as *V. nakaiana* Ohwi 1938 (Choi et al. 2016, Lee et al. 2021), *V. ovata* subsp. *kiusiana* Albach 2008 (Maurya et al. 2020), and *V. ovata* subsp.* kiusiana* var. *diamantiaca* (Ha et al. 2025), have been assembled in recent years, the complete chloroplast genomes of *V. longifolia* and *V. pusanensis* have not yet been reported. Understanding the genetic makeup of these native species is vital for developing new varieties as garden materials, supporting future plant breeding and germplasm utilization strategies (Kim and Cho 2024).

To address this critical gap and contribute to the understanding of this complex genus, we aimed to sequence, assemble, and characterize the complete chloroplast genomes of these two key *Veronica* species, *V. longifolia* and *V. pusanensis*. Our findings provide essential genomic resources to clarify the phylogenetic relationships within *Veronica* subgenus *Pseudolysimachion* and contribute to the broader taxonomic resolution of the tribe Veroniceae.

## Materials and methods

### Plant material, DNA extraction, and sequencing

**Figure 1.**
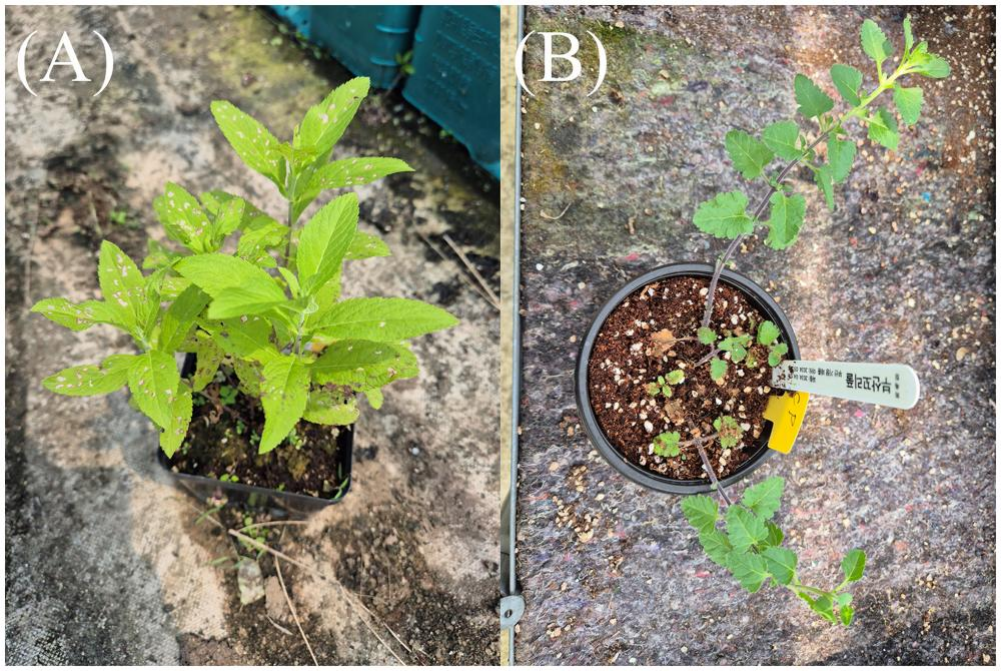
Photographs of Long-leaf spike speedwell (*Veronica longifolia*) (A) and Busan spike speedwell (*Veronica pusanensis*) (B) used for this study prior to sampling. The sample numbers are FBRUC2025SHK00A and FBRUC2025SHK00D, respectively (photos taken by Sang Heon Kim at a greenhouse at the Forest Biological Resources Utilization Center of the Korea National Arboretum).

*V. longifolia* (Voucher KHB1663331) was collected from Gurye-gun, Jeollanam-do, Korea (35°17’37.1"N 127°31’34.6"E), and *V. pusanensis* (Voucher KHB1663328) was collected from Gijang-gun, Busan, Korea (35°13’52.7"N 129°14’40.2"E). The determination of *V. longifolia* was confirmed using the comprehensive identification keys provided in Flora of Korea (Park 2018). The collected plants were propagated at the Forest Biological Resources Utilization Center of the Korea National Arboretum (Figure 1). Specimens were deposited at the Korea National Arboretum (Dr. Dong Chan Son, sdclym@korea.kr) with voucher numbers KHB1663331 and KHB1663328, respectively. Leaves of each plant were sampled and genomic DNA was extracted using HiGene™ Genomic DNA Prep Kit (Biofact, Daejeon, Korea) following the manufacturer’s protocols. The purified gDNA was sequenced using a NovaSeq6000 platform (Illumina, San Diego, CA, USA) by Biofact (Korea). Raw reads were quality-filtered and trimmed using Trimmomatic v0.39 to remove adapters and low-quality bases (quality score < 30). The mean read length of the paired-end reads was 150 bp. NOVOPlasty v4.3.5 was applied to construct the chloroplast genome sequence with a k-mer value of 27 and the default seed settings (Dierckxsens et al. 2017). The *rbcL* gene from *V. nakaiana* (NCBI Reference Sequence NC_031153) was used as the seed file for assembly. To validate the assembly, a read-mapping step was performed by mapping the trimmed paired-end reads back to the assembled cp genomes using BWA-MEM. The mapping confirmed the circular structure, and specific attention was paid to reads mapping across the IR/SC boundaries to confirm the quadripartite structure. The chloroplast genome sequence was annotated with GeSeq (Tillich et al. 2017). Circular maps of the chloroplast genomes were described using OGDRAW (Greiner et al. 2019).

### Phylogenetic analysis

To deduce the phylogenetic position of *V. longifolia* and *V. pusanensis*, 15 other complete chloroplast genome sequences were collected from NCBI GenBank, including *Lagotis yunnanensis* W.W.Sm. 1919 as an outgroup. Protein-coding gene extraction and concatenated sequencing were conducted using PhyloSuite v.1.2.2 (Zhang et al. 2020), and sequence alignment was performed using the MAFFT v7.313 plugin (Katoh and Standley 2013) in PhyloSuite. To establish the phylogenetic relationships, a maximum likelihood (ML) analysis was performed using the MEGA 12 (Kumar et al. 2024) with 1,000 bootstrap reiterations based on the GTR+I model, which was identified as the optimal evolution model.

## Results

### General characteristics of the chloroplast genomes

**Figure 2.**
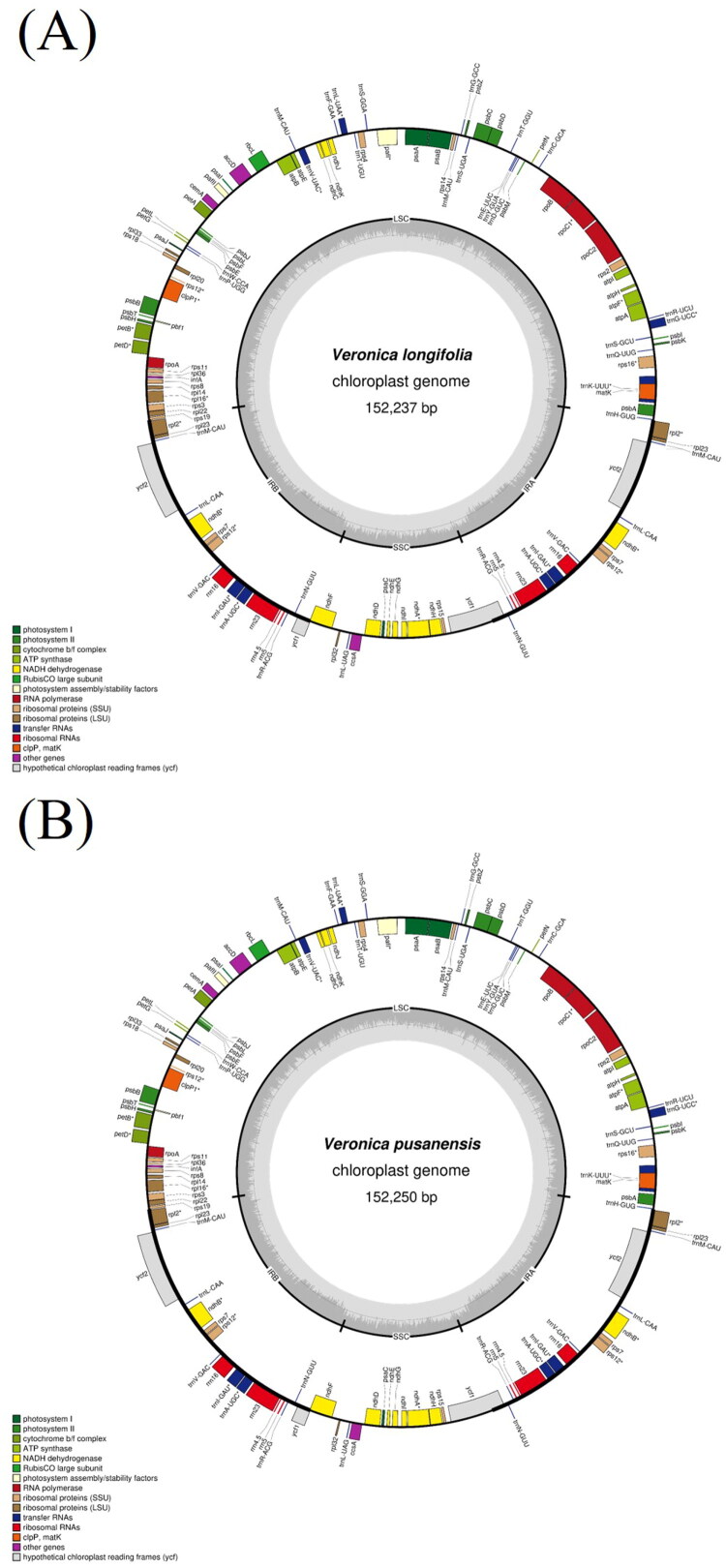
Complete chloroplast genome map of Long-leaf spike speedwell (*Veronica longifolia*) (A) and Busan spike speedwell (*Veronica pusanensis*) (B). Genes shown on the outside of the circle are transcribed clockwise, while those on the inside are transcribed counterclockwise. The inner circle displays GC content (dark gray) and AT content (light gray). The large single-copy (LSC) and small single-copy (SSC) regions are separated by inverted repeat A (IRA) and inverted repeat B (IRB) regions.

The complete cp genome sequence of *Veronica longifolia* (GenBank accession no. PX049137) and *Veronica pusanensis* (GenBank accession no. PX049138) were 152,237 bp and 152,250 bp in length, respectively (Figure 2, Table 1). Both genomes showed typical quadripartite structure including LSC, SSC, and a pair of IRs. The LSC region was 83,179 bp in *V. longifolia* and 83,183 bp in *V. pusanensis*, the SSC region was 17,700 bp and 17,697 bp, and the IR regions were 25,679 bp and 25,685 bp, respectively.

**Table 1.** Comparison of general characteristics of *veronica longifolia* and *veronica pusanensis.*

	*Veronica longifolia*	*Veronica pusanensis*
Sequencing information (Pair-end reads)		
Raw data read number	23,071,973	17,006,190
Mapped read number	2,697,771	8,737,569
Chloroplast coverage (×)	2643.98	8607.49
Chloroplast genome size (bp)		
Total genome	152,237	152,250
Large single-copy	83,179	83,183
Small single-copy	17,700	17,697
Inverted repeat	25,679	25,685
GC content (%)		
Total genome	38.0	38.0
LSC	36.1	36.1
SSC	31.9	31.9
IR	43.2	43.2
Number of genes		
Total	133	133
Protein-coding	88	88
tRNA	37	37
rRNA	8	8

The sequencing depth for *V. longifolia* was 2643.98× and for *V. pusanensis* was 8607.49× (Table 1, Figure S1). This difference primarily reflects the higher proportion of chloroplast reads in the total sequenced DNA for the *V. pusanensis* sample. For both chloroplast genomes, the overall GC content was 38.0%. Specifically, the LSC regions showed 36.1% GC content, the IR regions 43.2%, and the SSC regions 31.9%. Genome sizes and GC content are highly conserved across the compared *Veronica* subgenus *Pseudolysimachion* species, consistent with patterns in other Plantaginaceae plastomes. Both chloroplast genomes accommodated 133 genes in total, comprising 88 protein-coding genes (PCGs), 37 transfer RNA (tRNA) genes, and 8 ribosomal RNA (rRNA) genes. In both species, 13 PCGs were recognized as *cis-*splicing genes (Figure S2), while the *rps12* gene was identified as a *trans*-splicing gene (Figure S3).

### Phylogenetic analysis

**Figure 3.**
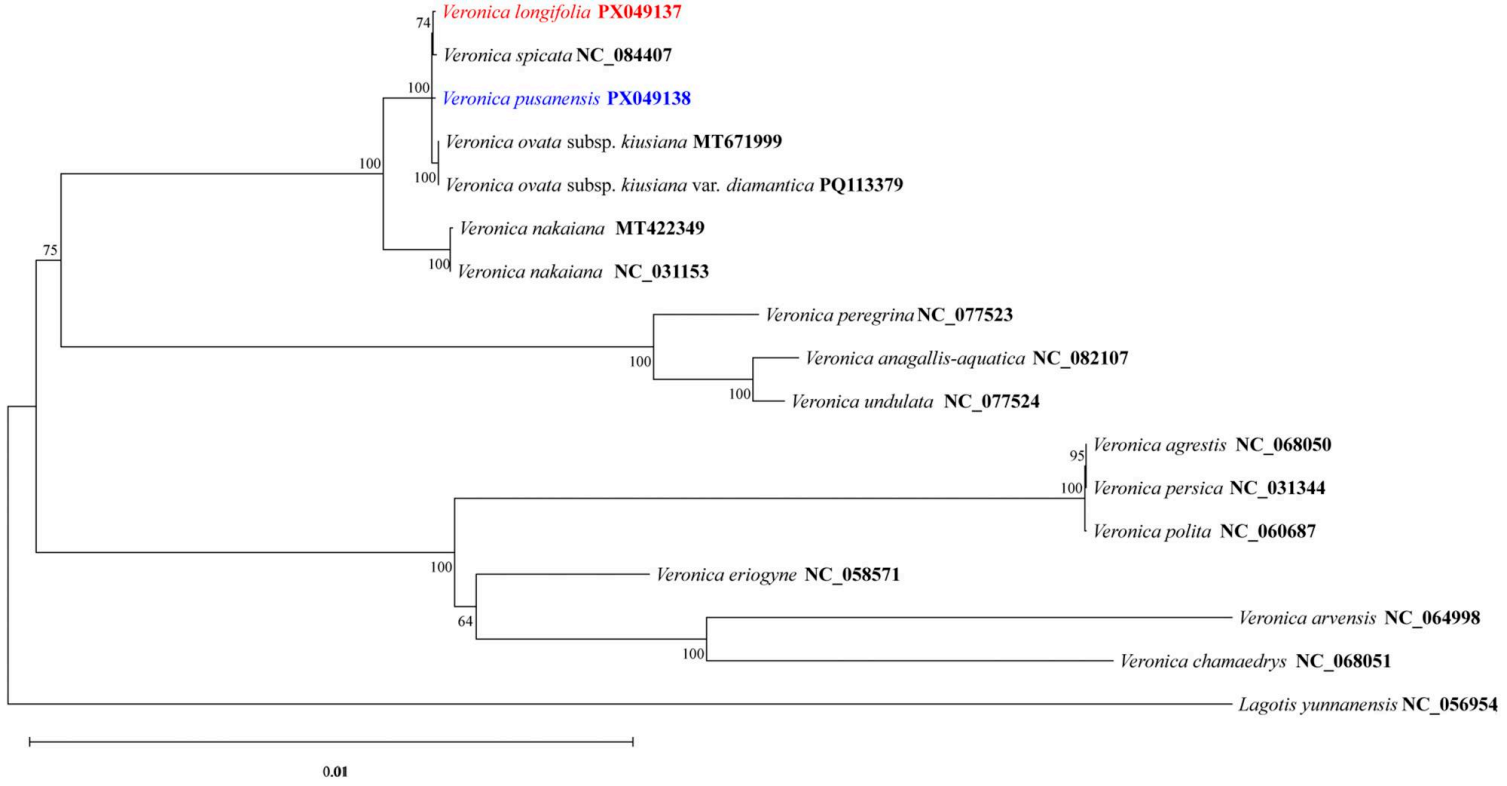
Maximum likelihood (ML) phylogenetic tree of the tribe veroniceae based on complete chloroplast genome sequences. Bootstrap support values (>50%) are shown at each node. *Veronica longifolia* PX049137 and *veronica pusanensis* PX049138, newly sequenced in this study, are highlighted in red and blue, respectively. The following sequences were used: *Veronica spicata* NC_084407, *Veronica ovata* subsp. *kiusiana* MT671999 (Maurya et al. 2020), *Veronica ovata* subsp. *kiusiana* var. *diamantiaca* PQ113379 (Ha et al. 2025), *Veronica nakaiana* MT422349 (Lee et al. 2021) and NC_031153 (Choi et al. 2016), *Veronica peregrina* NC_077523, *Veronica anagallis-aquatica* NC_082107 (Hai et al. 2024), *Veronica undulata* NC_077524, *Veronica agrestis* NC_068050 (Zhao et al. 2023), *Veronica persica* NC_031344 (Choi et al. 2016), *Veronica polita* NC_060687 (Jia et al. 2022), *Veronica eriogyne* NC_058571 (Danzeng et al. 2021), *Veronica arvensis* NC_064998 (Liu et al. 2022), *Veronica chamaedrys* NC_068051 (Zhao et al. 2023). *Lagotis yunnanensis* NC_056954 (Cheng et al. 2020) was used as an outgroup. The scale bar indicates the number of nucleotide substitutions per site.

The ML phylogenetic tree was constructed to elucidate the phylogenetic relationships within the tribe Veroniceae (Figure 3). The resulting ML tree strongly supported the monophyly of the Plantaginaceae family (BS = 100). The tribe Veroniceae, represented by these 16 taxa (excluding the outgroup *Lagotis yunnanensis*), was clearly resolved. Crucially, the genus *Veronica* was revealed to be highly polyphyletic in the context of the *Pseudolysimachion* samples, confirming that the *Pseudolysimachion* species form a monophyletic clade that is nested deeply within *Veronica*, a finding consistent with robust recent molecular studies (Albach 2008, Albach et al. 2004). Our analysis explicitly refutes the notion of *Pseudolysimachion* as a sister group to the entire *Veronica* genus. Within the *Veronica* subgenus *Pseudolysimachion* clade, *V. longifolia* formed a well-supported clade (BS = 74) with *V. spicata*. The Korean endemic species, *V. pusanensis*, was robustly grouped with *V. ovata* subsp. *kiusiana* and *V. ovata* subsp. *kiusiana* var. *diamantiaca* (BS = 100), but was positioned as a sister group to the *V. ovata* subsp. *kiusiana*/*V. ovata* subsp. *kiusiana* var. *diamantiaca* clade, not nested between them.

## Discussion and conclusion

Our study provides key insights into the phylogenetic relationships within the tribe Veroniceae, focusing on the classification of *Veronica* subgenus *Pseudolysimachion*. The Maximum Likelihood (ML) phylogenetic tree strongly supported the monophyly of the family Plantaginaceae (Albach et al. 2004, Maurya et al. 2020).

Crucially, our analysis utilized the newly assembled chloroplast genomes of *V. longifolia* and *V. pusanensis* to confirm the monophyly of the *Veronica* subgenus *Pseudolysimachion* clade. Our results align with the modern classification of *Veronica*, clearly showing that *Pseudolysimachion* species are not sister to *Veronica* but are nested deeply within it, consistent with comprehensive phylogenetic studies based on molecular data (Albach 2008, Albach et al. 2004). Furthermore, *V. longifolia* formed a well-supported clade with *V. spicata.* The Korean endemic species *V. pusanensis* grouped robustly with *V. ovata* subsp. *kiusiana* and *V. ovata* subsp. *kiusiana* var. *diamantiaca*, but its phylogenetic position as sister to the latter clade supports its distinction and may reflect historical biogeographic exchanges across the East Asian floristic region. These findings provide essential genomic resources that clarify the taxonomic positions of these species and contribute to our understanding of speciation patterns within the genus.

Although this study relied solely on chloroplast genome data, providing a solid foundation, future research should incorporate a broader sampling of *Veronica* species and low-copy nuclear gene data. As chloroplast genomes represent a single, maternally inherited locus, they may not reflect species trees if hybridization or incomplete lineage sorting are present. Incorporating nuclear markers would allow for the construction of a higher-resolution phylogenetic tree, offering deeper insights into interspecific divergence and the biogeographical history of this diverse genus on the Korean Peninsula.

## Supplementary Material

Supplementary Figures_20260102.docx

## Data Availability

The data obtained from this study are openly available in GenBank of NCBI at https://www.ncbi.nlm.nih.gov/ under the accession number PX049137 and PX049138. The associated BioProject, SRA, and BioSample numbers are PRJNA1295692, SRR34716176 and SRR34716177, and SAMN50182493 and SAMN50182797, respectively.
